# Antioxidant and physiological effects of *Si*-*Wu*-*Tang* on skin and liver: a randomized, double-blind, placebo-controlled clinical trial

**DOI:** 10.1186/s13020-016-0102-0

**Published:** 2016-06-30

**Authors:** Hui-Fang Chiu, Ying-Hua Wu, You-Cheng Shen, Shing-Jung Wang, Kamesh Venkatakrishnan, Chin-Kun Wang

**Affiliations:** Department of Chinese Medicine, Taichung Hospital Ministry of Health and Well-being, Taichung, Taiwan; School of Nutrition, Chung Shan Medical University, Taichung, Taiwan; School of Health Diet and Industry Management, Chung Shan Medical University, Taichung, Taiwan; Division of Research and Development, Standard Foods Corporation, Taipei, Taiwan

## Abstract

**Background:**

*Si*-*Wu*-*Tang* (SWT) is used to treat various gynecological disorders in Chinese medicine. This study investigated the antioxidant and physiological effects of SWT on the skin and liver in healthy adults.

**Methods:**

This randomized, crossover, double-blind, placebo-controlled clinical trial was conducted at Chung Shan Medical University Hospital in December 2008. Participants with uncontrolled diabetes, heart disease, liver disease, kidney disease, cancer, and pregnancy were excluded. Sixty healthy volunteers taking no medications were recruited from the community based on the results of their medical history questionnaires and biochemical analyses to confirm their health status. The participants were assigned to two groups: one group drank 125 mL of placebo (n = 30) and the other drank SWT (n = 30) for six continuous days per month for 6 months. The placebo and SWT were then switched between the groups after a 1-month washout period. Anthropometric measurements (body weight, body fat, and body mass index) were performed and fasting blood samples were drawn for various biochemical assays at 1, 3, 6, 10 and 13 months. Abdominal ultrasound and skin examinations were performed at 1, 6 and 13 months. The skin examinations involved assessment of skin roughness, sebum content, hydration, surface water loss, erythema, melanin index, and elasticity on the face (sunlight-exposed sites: middle of ear and nose) and inner arm (sunlight-unexposed sites: center of wrist and elbow joint).

**Results:**

Administration of SWT significantly increased the antioxidant index (*P* = 0.001) and antioxidant enzymes activities (*P* = 0.001) from baseline to month 6. SWT also suppressed the concentration of serum lipids (triglycerides, *P* = 0.01; high-density lipoprotein cholesterol, *P* = 0.23; low-density lipoprotein cholesterol, *P* = 0.48) and hepatic marker enzymes (glutamic pyruvic transaminase, *P* = 0.76; glutamic oxaloacetic transaminase, *P* = 0.65) when compared with the placebo group. Abdominal ultrasound in the SWT group revealed a positive impact of SWT on mild fatty liver, gallstones, and mild splenomegaly. Moreover, SWT intake concomitantly elevated erythema (*P* = 0.011) and markedly lowered skin surface water loss (*P* = 0.016), sebum content (*P* = 0.021), and wrinkles (*P* = 0.024).

**Conclusions:**

Oral administration of SWT for 6 months improved the antioxidant level and positively regulated the lipid profile, liver function, and skin integrity and texture.

**Electronic supplementary material:**

The online version of this article (doi:10.1186/s13020-016-0102-0) contains supplementary material, which is available to authorized users.

## Background

The therapeutic essence of Chinese medicine (CM) can be attributed to multiple elements rather than just one in the product used [1]. *Si*-*Wu*-*Tang* (SWT) is a CM formula comprising four different medicinal herbs: *Radix Paeoniae Alba* (*bai shao yao*), *Rhizoma* Ligusticum *Chuanxiong* (*chuan xiong*), *Radix Angelica Sinensis* (*dang gui*), *and Radix Rehmanniae Preparata* (*shu di* huang) [2]. SWT is widely used for the treatment of gynecological disorders such as menstrual discomfort, climacteric syndrome, dysmenorrheic and other estrogen-related diseases [3], cutaneous diseases [4], and menstrual discomfort [5]. Several preclinical (in vitro) studies of SWT have demonstrated antioxidative, anti-inflammatory, antiaging, antibacterial, and antipruritic activities [6, 7].

The major bioactive components in these four herbs include phenolics, phthalides, alkaloids, terpene glycosides, and iridoid glycosides. Some major constituents are gallic acid, paeoniflorin, senkyunolide A, ferulic acid, Z-ligustilide, butylphthalide, sodium ferulate, and catalpol [3]. Among these compounds, ferulic acid, paeoniflorin, and Z-ligustilide in SWT exhibited antioxidative, antimutagenic, anti-inflammatory, vasodilative, and antiallergic effects [8–10]. Sodium ferulate in *A. sinensis* and *Lignsticum chuangxiong* of SWT has lowered the oxidation process and lipid levels in various animal models [11, 12]. Additionally, SWT is rich in organic acids, amino acids, polysaccharides, vitamins, and minerals such as magnesium, calcium, phosphorus, and iron [13].

Liver cells (hepatocytes) metabolize toxins such as alcohol and drugs into their inactive metabolites. Hepatocytes are extremely prone to peroxide (free radical) formation because of their higher metabolic rate. These free radicals are effectively eliminated by antioxidants, especially glutathione peroxidase (GPx). GPx plays a major role in the reductive detoxification of peroxides in hepatocytes [14]. A decreased antioxidant level may lead to oxidative stress and eventual hepatic dysfunction. Several reports have proven that hepatic dysfunction might contribute to various dermal disorders such as dermatitis, eczema, psoriasis, acne, and boils or rashes secondary to decreased detoxification (excessive toxin buildup in skin) and elevated inflammation and oxidative stress [15, 16]. Hence, this study was designed to investigate the antioxidant and physiological effects of SWT on skin and liver in healthy adults.

## Methods

### Chemicals

Folin–Ciocalteu phenol reagent, sodium hydroxide, sodium nitrite, hydrochloric acid, 6-hydroxy-2,5,7,8-tetramethylchromane-2-carboxylic acid (Trolox), hydrogen peroxide, sodium dihydrogen phosphate, and trichloroacetic acid were purchased from Sigma (St. Louis, MO, USA).

### SWT and placebo

Commercially available SWT and placebo as an oral suspension (glass vial) were provided by Standard Foods Corporation (Taipei, Taiwan). Each vial contained 125 mL of SWT (*Radix P. Alba*, *Rhizoma L. Chuanxiong*, *Radix A. Sinensis*, and *Radix R. Preparata* with cinnamon twig, rose hip extract, sucrose, vitamin C, malic acid, citric acid, and gelatin) or placebo (simulated SWT flavor with herb-rinsed water and sucrose solution). Both sample vials were indistinguishably packaged with a similar color, flavor, size, and shape.

### Participants

This randomized, crossover, double-blind, placebo-controlled clinical trial was conducted in Chung Shan Medical University Hospital. The trial was carried out in accordance with the declaration of Helsinki and subsequent revisions and approved by the Institutional Review Board (IRB) of Chung Shan Medical University Hospital, Taichung, Taiwan (CSMUHCS08008) and registered with clinicaltrials.gov (NCT02634242) (Additional file 1). Written informed consent was received from all participants before enrollment (Additional file 2). Taking into account an expected dropout rate of approximately 25 % and the crossover design, the required sample size (95 % CI, α = 0.05) was 60 participants (30 participants in each group) [17]. All participants (aged 20–80 years) were enrolled in the present experiment via advertisements displayed in public places.

The participants were requested to avoid using medications or supplements during the intervention. They could withdraw from the study at any time if desired. The exclusion criteria were a history of smoking, alcoholism, pregnancy or lactation, chronic diseases, and hepatic or renal dysfunction. A physical examination was performed in the 60 voluntary participants at the beginning of the study, and they were segregated into two groups of 30 participants in each (five male and 25 female): those who drank 125 mL of placebo and those who drank SWT for 6 days per month for 6 months. The placebo and SWT were switched between the two groups after a 1-month washout period. Anthropometric analyses of body weight, body fat, and body mass index were performed at 1, 3, 6, 10 and 13 months. The SWT and placebo vials were labeled with the participant number by an electrical randomization method [17]. The average percentage intake of SWT or placebo was 88.47 % at the end of the experiment based on the participants’ records. During the crossover study, three and two female participants in the placebo and SWT groups, respectively, were excluded from the study because of an unwillingness to cooperate. Thus, the present study was performed with 27 and 28 participants in the placebo and SWT group, respectively (*n* = 55).

### Blood sampling

At baseline (months 1 and 7), the middle time point (months 3 and 10), and the end of each intervention period (months 6 and 13), fasting blood samples were collected in an EDTA-coated vacuum tube, and plasma was separated by centrifugation (Supercentrifuge 1K15; Sigma) at 1500×*g*. Blood samples were isolated after separating the intermediate film, and the settled portion was washed with isotonic saline and centrifuged at 1500×*g* to obtain the erythrocytes for assaying antioxidant enzymes. All samples were stored at −80 °C until analyses.

Abdominal ultrasonic examination was performed at month 1, 6 and 13 at Chung Shan Medical University Hospital, Taichung, Taiwan with the help of an experienced gastroenterology physician (Dr. Chen Ziyan).

### Various oxidative indexes

The total antioxidant capacity (TEAC) [18, 19], total thiobarbituric acid reactive substances (TBARS) [20], and glutathione content [21] in plasma were determined by previously reported methods.

### Antioxidative enzymes

Superoxide dismutase (SOD) activity was determined with the Ransod SD 125 kit (Randox Labs, Crumlin, UK), GPx and glutathione reductase (GR) activity were determined with the Ransel RS 504 kit (Randox Labs, Crumlin, UK), and catalase (CAT) activity was measured by Aebi [22]. The red blood cell protein content was determined based on the biuret reaction of the BCA kit from Thermo Fisher Scientific (MA, USA).

### Serum lipids and hepatic markers

The serum concentrations of total cholesterol (TC), triglycerides (TG), and high-density lipoprotein cholesterol (HDL-c) were measured by commercially available lipid profile kits (CHODPAP for TC, GPO-PAP for TG, and a precipitation method for HDL-c; Roche Diagnostics, Mannheim, Germany) on a Hitachi 747 autoanalyzer. Low-density lipoprotein cholesterol (LDL-c) was calculated by the Friedewald equation. The plasma glutamic oxaloacetic transaminase (GOT) and glutamic pyruvic transaminase (GPT) concentrations were measured by AppliedBio assay kits (Hercules, CA, USA).

### Skin examination

A multifunctional skin detector (MPA 580; Courage and Khazaka Electronic GmbH, Cologne, Germany) and skin roughness analyzer (VD 300; Courage and Khazaka Electronic GmbH) were used to detect the biophysical characteristics of skin in the face and arm regions. No skin care products were applied to the measured sites for at least 24 h prior to the measurements. The skin sebum content, hydration, surface water loss, erythema, melanin index, and elasticity were measured with the following respective probes: Sebumeter, Corneometer, TEWAmeter, Mexameter, and Cutometer (Courage and Khazaka Electronic GmbH).

The Sebumeter SM 815 utilizes the difference in light intensity via a plastic strip to indicate the quantity of absorbed sebum. The sebum level is expressed as μg/cm^2^ [23]. Skin hydration was measured by Corneometer CM 825, which uses the high dielectric constant of water to evaluate the water-related changes in the electrical capacitance of the skin. It displays hydration measurements in system-specific arbitrary units [24]. A melanin index is calculated by the Mexameter MX 18 from the strength of the absorbed and reflected light at 660 and 880 nm, respectively. Erythema is processed similarly at 568 and 660 nm, respectively [25]. The measurement of cutaneous water loss by TEWAmeter TM 300 was analyzed based on the diffusion in an open chamber and is expressed as g/m^2^/h [26]. The Cutometer MPA 580 pulls the targeted skin into the probe with controlled vacuum pressure [27]. Skin wrinkles were measured using a Visioscan VC 98 (Courage and Khazaka Electronic GmbH). In brief, two points were measured: one at the middle of the nose and right ear, and the other at the middle of the wrist and inner elbow joint of right hand. All participants were instructed to wash and clean their face and arm, and then rested calmly for 30 min in a room kept at 20 ± 2 °C and a relative humidity of 50 ± 2 % before testing.

### Statistical analysis

The results are expressed as a mean ± standard deviation. The paired t test was employed to compare differences within the same group (baseline vs. end of treatment), and Student’s t test was used to compare differences between the experimental (SWT) and control (placebo) groups. All variables were analyzed via one-way analysis of variance with the *post doc* least-significant differences test. A *P* < 0.05 was considered statistically significant. The Statistical Package for the Social Sciences (SPSS) version 17.0 (Chicago, IL, USA) was used for analysis.

## Results and discussion

**Fig. 1:**
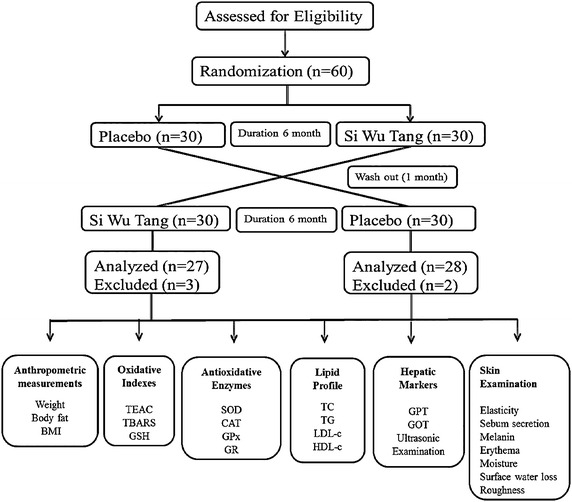
Flow chart of present study

A crossover design was chosen for the present investigation because of its numerous advantages such as decreased confounding covariants (because the same participants ingested both SWT and placebo) as well as statistically effective during chronic condition [28]. Based on our literature survey, a 1-month wash-out period was adopted for the present study to suppress the carry-over effect. A flowchart of the present study is illustrated in Fig. 1. The anthropometric parameters in the placebo and SWT groups are presented in Table 1. At baseline, the body weight, body fat, and body mass index were within the normal range in both the placebo and SWT groups. There were no significant differences in any of the anthropometric parameters between the placebo and SWT groups at the end of the intervention.

**Table 1:** The anthropometric parameters in placebo and SWT treated healthy subjects

Group	Weight (kg)	Body fat (%)	BMI (kg/m^2^)
Baseline			
Placebo	57.46 ± 9.43^a^	29.23 ± 5.82^a^	22.29 ± 3.18^a^
SWT	57.45 ± 9.58^a^	29.27 ± 5.77^a^	22.35 ± 3.19^a^
3rd month			
Placebo	57.74 ± 9.80^a^	29.47 ± 5.91^a^	22.39 ± 3.28^a^
SWT	57.70 ± 9.48^a^	29.15 ± 5.70^a^	22.44 ± 3.21^a^
6th month			
Placebo	57.70 ± 9.28^a^	29.32 ± 6.09^a^	22.32 ± 3.46^a^
SWT	57.41 ± 9.67^a^	29.25 ± 5.77^a^	22.07 ± 3.43^a^

Values were expressed as mean ± SD (*n* = 55). Data within the same column of each group sharing different superscript letters were significantly different (*p* < 0.05)

*BMI* body mass index

Antioxidants interact with free radicals and stabilize by donating their electrons or protons. If untreated, these free radicals can cause various chronic diseases [29]. Table 2 shows the plasma oxidative indexes including, TEAC, glutathione (GSH) concentration, and TBARS in the placebo- and SWT-treated participants. The TEAC reflects the relative ability of hydrogen or electron-donating antioxidants to scavenge the 2,2′-azino-bis 3-ethylbenzothiazoline-6-sulphonic acid (ABTS) radical cation compared with that of Trolox. Treatment with SWT for 6 months substantially increased the levels of TEAC (0.35–0.59 µM; *P* = 0.001) and GSH (14.19–14.35 µM; *P* = 0.88), whereas the TBARS level (1.40–0.68 µM; *P* = 0.001) was lower than that in the baseline period; additionally, notable changes were found between the SWT and placebo groups. SWT comprises four medicinal plants with numerous active components including gallic acid, paeoniflorin, ferulic acid, and Z-ligustilide, which contain numerous OH groups that scavenge free radicals through electron delocalization [6]. Liu et al. [30] reported that polysaccharide from *A. sinensis* could directly scavenge oxygen-derived free radicals in colonic tissues of rats with colitis.

**Table 2:** Various plasma oxidative indexes in placebo and SWT treated healthy subjects

Group	TEAC (µM/mL)	*p* value*	TBARS (μM/L)	*p* value*	GSH (μM/L)	*p* value*
Baseline						
Placebo	0.34 ± 0.07^a^	0.82	1.43 ± 0.47^a^	0.87	14.18 ± 2.30^b^	0.67
SWT	0.35 ± 0.07^b^		1.40 ± 0.44^a^		14.19 ± 2.48^b^	
3rd month						
Placebo	0.34 ± 0.04^a^	0.001	1.42 ± 0.43^a^	0.001	14.19 ± 2.79^a^	0.83
SWT	0.40 ± 0.07^b^		0.83 ± 0.28^b^		14.31 ± 2.72^a^	
6th month						
Placebo	0.35 ± 0.03^a^	0.001	1.45 ± 0.32^a^	0.001	14.16 ± 2.85^a^	0.88
SWT	0.59 ± 0.06^a^		0.68 ± 0.23^c^		14.35 ± 2.94^b^	

Values were expressed as mean ± SD (*n* = 55). Data within the same column of each group bearing different superscript letters were significantly different (*p* < 0.05)

* Student’s t test was used to assess statistical significance between placebo and Si Wu Tang (SWT)

The antioxidant defense system consists of endogenous antioxidants SOD, CAT, GPx, and GR. SOD catalyzes the dismutation of superoxide into hydrogen peroxide and water molecules. After the decomposition of hydrogen peroxide by GPx at the expense of GSH, the GR-catalyzed regeneration of GSH from its oxidized form can sustain the GSH-dependent oxy-radical scavenging activity [31].

The antioxidant enzymes evaluated in this study (SOD, CAT, GPx, and GR in the erythrocytes of the placebo- and SWT-treated participants) are epitomized in Table 3. No notable changes were found from baseline to month 6 in the placebo-treated group. However, treatment with 125 mL of SWT showed a marked escalation in the levels of SOD (1478.12–1812.47 IU/g Hb; *P* = 0.001), CAT (565.82–803.32 IU/g Hb; *P* = 0.001), GPx (51.03–52.59 IU/g Hb; *P* = 0.46), and GR (10.51–11.93 IU/g Hb; *P* = 0.02) between baseline and the end of the treatment (month 6). Meanwhile, significant changes were also noted between the SWT and placebo groups at month 6.

**Table 3:** Erythrocyte antioxidative enzymes in placebo and SWT treated healthy subjects

Group	SOD (IU/g Hb)	*p* value*	CAT (IU/g Hb)	*p* value*	GPx (IU/g Hb)	*p* value*	GR (IU/g Hb)	*p* value*
Baseline								
Placebo	1475.85 ± 155.91^a^	0.59	562.82 ± 91.86^a^	0.97	51.04 ± 8.61^a^	0.99	10.45 ± 1.69^a^	0.86
SWT	1478.86 ± 230.38^c^		565.13 ± 89.02^c^		51.02 ± 8.90^b^		10.54 ± 1.80^b^	
3rd month								
Placebo	1471.12 ± 183.53^a^	0.01	553.14 ± 66.28^a^	0.001	51.08 ± 6.99^a^	0.63	10.90 ± 1.98^a^	0.01
SWT	1572.01 ± 238.24^b^		677.61 ± 98.83^b^		51.82 ± 8.97^a^		11.94 ± 2.34^a^	
6th month								
Placebo	1475.67 ± 165.34^a^	0.001	569.36 ± 72.39^a^	0.001	51.61 ± 7.05^a^	0.46	10.86 ± 2.01^a^	0.02
SWT	1812.47 ± 343.74^a^		803.32 ± 93.10^a^		52.59 ± 6.86^ab^		11.93 ± 2.66^a^	

Values were expressed as mean ± SD (*n* = 55). Data within the same column of each group bearing different superscript letters were significantly different (*p* < 0.05)

* Student’s t test was used to assess statistical significance between placebo and Si Wu Tang (SWT)

Nuclear factor erythroid 2-related factor 2 (Nrf2) by Z-ligustilide was involved in the antioxidant activity in the SWT group. Activation of Nrf2 triggered the downstream antioxidant genes (SOD, CAT) and thereby enhanced antioxidant enzyme levels [32]. Additionally, the polyphenolics and numerous phytochemical constituents present in SWT might have elevated the levels of antioxidant enzymes by their free radical quenching ability [7]. Hou et al. [33] reported that treatment with *L. chuanxiong and A. sinensis* showed a better protection against hydrogen peroxide-induced endothelial damage by suppressing reactive oxygen species production. Furthermore, trace elements (calcium, magnesium, and selenium) in SWT act as cofactors to antioxidant enzymes [34].

Lipids are metabolized and regulated by hepatocytes. Any changes in the hepatic lipid status could directly reflect the serum lipid status. Although they are not major hepatic markers, lipids are still effective markers with which to determine whether SWT impacts physiological changes in hepatic tissue by estimating the lipid profile in serum. Table 4 shows the lipid profiles in the serum of the placebo and SWT groups. Supplementation with SWT for 6 months resulted in a significant decline in the levels of TG (102.12–85.89 mg/dL; *P* = 0.01) with a slight decrement in the level of LDL-c (113.05–112.84 mg/dL; *P* = 0.48), whereas the level of HDL-c was concomitantly improved when compared with the baseline value (57.52–59.13 mg/dL; *P* = 0.23). However, no marked changes were observed in the TC level. These outcomes indicate that SWT did not change the cholesterol levels; instead, it substantially changed the TG levels, probably owing to the presence of paeoniflorin in SWT. Paeoniflorin works as an inhibitor of pancreatic lipase and thereby lowers TG levels [35]. Our results are consistent with those of Li et al. [36], who concluded that oral administration of polysaccharides from *A. sinensis* significantly reduced the TG and LDL-c levels and improved the HDL-c level in STZ-induced diabetic rats. Moreover, sodium ferulate in *A. sinensis* and *L. chuangxiong* lowered lipid levels in various animal models [11, 12].

**Table 4:** The lipid profile in plasma of placebo and SWT treated healthy subjects

Group	TG (mg/dL)	*p* value*	TC (mg/dL)	*p* value*	HDL-c (mg/dL)	*p* value*	LDL-c (mg/dL)	*p* value*
Baseline								
Placebo	100.22 ± 25.60^a^	0.81	192.85 ± 38.05^a^	0.95	57.02 ± 11.21^a^	0.87	114.98 ± 32.99^a^	0.69
SWT	102.12 ± 28.60^a^		192.05 ± 35.34^a^		57.52 ± 10.25^b^		113.05 ± 31.23^a^	
3rd month								
Placebo	97.56 ± 14.96^a^	0.01	191.71 ± 34.63^a^	0.62	58.60 ± 12.88^a^	0.45	113.60 ± 34.04^a^	0.83
SWT	87.27 ± 14.64^b^		192.20 ± 35.55^a^		59.20 ± 13.41^a^		113.13 ± 32.32^a^	
6th month								
Placebo	97.64 ± 23.39^a^	0.01	192.36 ± 32.85^a^	0.88	57.51 ± 12.75^a^	0.23	113.84 ± 32.21^a^	0.48
SWT	85.89 ± 17.86^b^		192.73 ± 36.25^a^		59.13 ± 12.31^a^		112.84 ± 33.68^a^	

Values were expressed as mean ± SD (*n* = 55). Data within the same column of each group bearing different superscript letters were significantly different (*p* < 0.05)

* Student’s t test was used to assess statistical significance between placebo and Si Wu Tang (SWT)

The serum GOT and GPT activities are reliable indicators of liver injury [37]. The concentrations of these hepatic markers in the placebo and SWT groups of the present study are shown in Table 5. In a comparison of the baseline and end-of-intervention values in the SWT group, no significant changes were noted in the GOT concentration (20.67–20.33 U/L; *P* = 0.65), but a slight decrease was observed in the GPT concentration (19.00–18.51 U/L; *P* = 0.76). This indicates that SWT can improve hepatic function by lowering hepatic marker enzymes. Wang et al. [38] concluded that treatment with *Radix P. Rubra* and *Radix P. Alba* significantly attenuated CCl_4_-induced acute liver injury in a rat model. *Angelica sinensis* exhibited a hepatoprotective effect against hepatotoxicity induced by CCl_4_ attributing to its antioxidant activity [39].

**Table 5:** The hepatic marker enzymes in plasma of placebo and SWT treated healthy subjects

Group	GPT (U/L)	*p* value*	GOT (U/L)	*p* value*
Baseline				
Placebo	19.05 ± 0.27^a^	0.92	20.61 ± 6.37^a^	0.91
SWT	19.00 ± 0.93^a^		20.67 ± 6.46^a^	
3rd month				
Placebo	19.27 ± 0.46^a^	0.68	20.11 ± 6.63^a^	0.72
SWT	19.44 ± 0.94^b^		20.35 ± 7.74^a^	
6th month				
Placebo	19.47 ± 0.79^a^	0.76	21.42 ± 7.17^a^	0.65
SWT	19.56 ± 15.11^b^		21.16 ± 7.83^a^	

Values were expressed as mean ± SD (*n* = 55). Data within the same column of each group bearing different superscript letters were significantly different (*p* < 0.05)

* Student’s t test was used to assess statistical significance between placebo and Si Wu Tang (SWT)

**Fig. 2:**
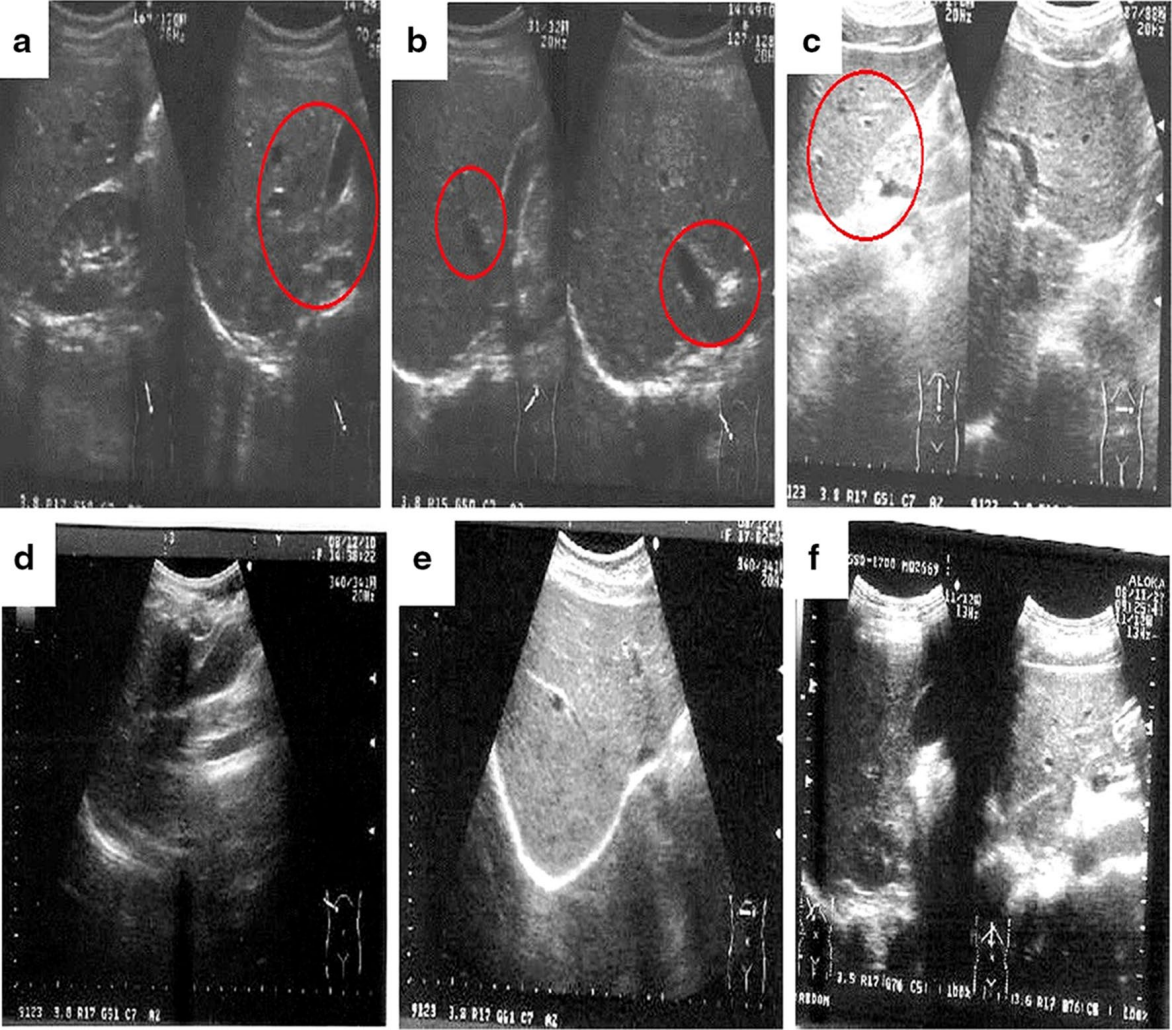
The Abdominal ultrasonic image of SWT treated healthy participants. **a**–**c** represented participant no. 3, 9 and 28 at baseline with mild fatty liver, gall bladder stone and splenomegaly respectively (indicated with *arrow mark*). **d**–**f** represents participant no. 3, 9 and 28 at 6th month of SWT treatment with normal inference (no signs of fatty liver, gall bladder stone and splenomegaly)

The abdominal ultrasonic examination findings in the placebo- and SWT-treated participants are shown in Fig. 2. The examination findings revealed mild fatty liver (Fig. 2a), gallbladder stones (Fig. 2b), and splenomegaly (Fig. 2c) in participant nos. 3, 9 and 28, respectively, at baseline. Treatment with SWT for 6 months in these participants resulted in normal examination findings with no signs of fatty liver (Fig. 2d), gallbladder stones (Fig. 2e), or splenomegaly (Fig. 2f). SWT improved hepatic function by reducing reactive oxygen species, thereby lowering the lipid profile parameters and hepatic damage as well as normalizing various abnormal conditions such as fatty liver, gallbladder stones, and splenomegaly owing to its free radical quenching activity.

The skin plays a crucial role in protecting the body from physical and chemical factors and moisture loss [40]. As discussed earlier, the liver is a major metabolic organ involved in detoxification with the help of GSH, GPx, GST, and GR. Decreases in the antioxidant levels in liver may lead to hepatic dysfunction. Several reports have proven that hepatic dysfunction can lead to various dermal disorders such as eczema, psoriasis, acne, boils, or rashes [15, 16]. The present study investigated the physiological correlation between hepatic and dermal organs with respect to antioxidation in healthy subjects.

Skin elasticity, moisture, surface water loss, sebum content, melanin, erythema, and wrinkles were analyzed to determine skin changes in the face and arm regions. Table 6 shows the results of the skin examination in the placebo and SWT groups. In the placebo group, no significant changes were noted in any of the skin parameters. No marked changes in skin elasticity, moisture, or melanin content were observed between baseline and 6 months in the SWT group; however, a significant increment in erythema was noted in the facial skin (261.12–275.55), whereas the skin surface water loss (5.90–4.87 g/m^2^/h), sebum content (163.86–144.96 μg/cm^2^), and skin wrinkles (37.41–35.23) in the arm were substantially lower than those at baseline.

**Table 6:** Skin examination in placebo and SWT treated all healthy subjects

Parameters	Duration	Placebo		SWT	
		Face	Arm/T-zone	Face	Arm/T-zone
Skin elasticity	Baseline	0.806 ± 0.09^a^	0.885 ± 0.08^a^	0.806 ± 0.09^a^	0.883 ± 0.05^a^
	6th month	0.805 ± 0.08^a^	0.874 ± 0.05^a^	0.814 ± 0.08^a^	0.805 ± 0.08^a^
Skin moisture	Baseline	45.18 ± 10.75^a^	49.45 ± 9.66^a^	45.18 ± 10.75^a^	49.45 ± 9.66^a^
	6th month	45.20 ± 9.86^a^	49.40 ± 10.83^a^	45.11 ± 8.62^a^	49.08 ± 9.49^a^
Surface water loss (g/m^2^/h)	Baseline	9.43 ± 1.07^a^	5.90 ± 0.66^a^	9.43 ± 1.07^a^	5.90 ± 2.36^a^
	6th month	9.34 ± 1.54^a^	5.52 ± 1.79^a^	9.14 ± 3.73^a^	4.87 ± 1.61^b^
Sebum (μg/cm^2^) content	Baseline	71.46 ± 6.42^a^	163.86 ± 26.46^a^	71.46 ± 6.42^a^	163.86 ± 26.46^a^
	6th month	64.02 ± 5.34^a^	152.76 ± 28.37^a^	62.06 ± 6.22^a^	144.96 ± 22.95^b^
Skin melanin index	Baseline	191.47 ± 40.59^a^	185.76 ± 28.02^a^	191.47 ± 40.59^a^	185.76 ± 31.02^a^
	6th month	193.68 ± 34.78^a^	185.22 ± 28.78^a^	190.95 ± 41.44^a^	184.91 ± 40.51^a^
Skin erythema index	Baseline	261.12 ± 49.07^a^	227.68 ± 35.96^a^	261.12 ± 79.07^a^	227.68 ± 55.96^a^
	6th month	266.37 ± 47.55^a^	229.40 ± 34.88^a^	275.55 ± 72.11^b^	239.06 ± 57.14^a^
Skin wrinkles	Baseline	34.84 ± 2.82^a^	37.41 ± 3.89^a^	34.84 ± 2.82^a^	37.41 ± 3.89^a^
	6th month	34.97 ± 3.42^a^	37.02 ± 3.04^a^	34.06 ± 5.70^a^	35.23 ± 3.20^b^

Values were expressed as mean ± SD (*n* = 55). Data within the same column of each group sharing different superscript letters (^a, b^) were significantly different (*p* < 0.05)

Table 7 shows the skin examination results in the placebo- and SWT-treated participants with and without a menstrual cycle. In the placebo group, no significant changes were noted in any of the skin parameters. No significant changes in skin elasticity, moisture, or melanin content from baseline to 6 months were observed in the SWT-treated group in either the face or arm region. A notable elevation in erythema was noted in the facial skin (295.57–303.66; *P* = 0.031) in participants without a menstrual cycle; however, participants with a menstrual cycle showed elevated erythema in both the facial (238.16–256.80; *P* = 0.022) and arm skin (206.65–222.97; *P* = 0.021). The skin surface water loss (5.97–5.06 g/m^2^/h; *P* = 0.025), sebum content (164.91–138.00 μg/cm^2^), and skin wrinkles (37.22–34.95; *P* = 0.032) in the arms of participants with a menstrual cycle were substantially lower than those of baseline.

**Table 7:** Skin examination in placebo and SWT treated healthy subjects with and without menstrual cycle

Parameters	Duration	Placebo		Si Wu Tang (SWT)			
				Without menstrual cycle		With menstrual cycle	
		Face	Arm/T-zone	Face	Arm/T-zone	Face	Arm/T-zone
Skin elasticity	Baseline	0.806 ± 0.09^a^	0.883 ± 0.05^a^	0.774 ± 0.08^a^	0.856 ± 0.06^a^	0.827 ± 0.09^a^	0.901 ± 0.05^a^
	6th month	0.805 ± 0.08^a^	0.874 ± 0.05^a^	0.769 ± 0.08^a^	0.864 ± 0.06^a^	0.843 ± 0.06^a^	0.895 ± 0.06^a^
Skin moisture	Baseline	45.18 ± 10.75^a^	49.45 ± 9.66^a^	44.83 ± 10.75^a^	47.42 ± 9.20^a^	45.41 ± 10.40^a^	50.80 ± 9.86^a^
	6th month	45.20 ± 9.86^a^	49.40 ± 10.83^a^	48.11 ± 8.62^a^	51.08 ± 9.94^a^	43.11 ± 8.62^a^	47.45 ± 9.09^a^
Surface water loss (g/m^2^/h)	Baseline	9.43 ± 1.07^a^	5.90 ± 0.66^a^	10.02 ± 1.90^a^	5.81 ± 0.98^a^	9.04 ± 1.07^a^	5.97 ± 1.89^a^
	6th month	9.34 ± 1.54^a^	5.52 ± 0.79^a^	9.83 ± 3.26^a^	4.59 ± 1.08^a^	9.34 ± 3.73^a^	5.06 ± 1.55^b^
Sebum (μg/cm^2^) content	Baseline	71.46 ± 6.42^a^	152.86 ± 26.46^a^	68.82 ± 6.53^a^	162.27 ± 16.46^a^	73.24 ± 6.42^a^	164.91 ± 16.46^a^
	6th month	64.02 ± 5.34^a^	163.76 ± 28.37^a^	58.00 ± 6.96^a^	155.41 ± 30.47^a^	71.42 ± 6.22^a^	138.00 ± 22.95^b^
Skin melanin index	Baseline	191.47 ± 40.59^a^	185.76 ± 28.02^a^	201.02 ± 40.59^a^	204.27 ± 31.02^a^	185.10 ± 40.59^a^	173.41 ± 27.43^a^
	6th month	193.68 ± 34.78^a^	185.22 ± 28.78^a^	202.80 ± 41.44^a^	203.29 ± 40.51^a^	183.05 ± 41.44^a^	172.65 ± 30.51^a^
Skin erythema index	Baseline	261.12 ± 49.07^a^	227.68 ± 35.96^a^	295.57 ± 49.07^a^	259.23 ± 64.10^a^	238.16 ± 54.70^a^	206.65 ± 38.06^a^
	6th month	266.37 ± 47.55^a^	229.40 ± 34.88^a^	303.66 ± 41.17^b^	263.21 ± 64.58^a^	256.80 ± 59.54^b^	222.97 ± 45.88^b^
Skin wrinkles	Baseline	34.84 ± 2.82^a^	37.41 ± 3.89^a^	35.66 ± 3.15^a^	37.69 ± 4.96^a^	34.30 ± 2.48^a^	37.22 ± 3.05^a^
	6th month	34.97 ± 3.42^a^	37.02 ± 3.04^a^	34.83 ± 3.03^a^	36.66 ± 3.92^a^	34.54 ± 3.61^a^	34.95 ± 2.65^b^

Values were expressed as mean ± SD (*n* = 55). Data within the same column of each group sharing different superscript letters (^a, b^) were significantly different (*p* < 0.05)

SWT increased the blood circulation to maintain hemostasis via stimulation of hematopoietic stem cells [41] and thus increased the immune response and elevated the erythema condition. The elevation in the blood cell concentration may alter various hormone levels (e.g., antidiuretic hormone), which may decrease the skin surface water loss, control water retention, and stabilize collagen production, thus decreasing skin wrinkles with elevated antioxidation activity on the skin surface of SWT-treated participants. Because SWT eases menstrual cycle-related dysfunction, it may also boost blood circulation throughout the body and hence decrease skin-related dysfunctions by flushing out toxins in the skin. However, we also encountered a controversial result showing decreased sebum secretion in SWT-treated participants with a menstrual cycle. Ohta et al. [42] reported that the skin sebum content was reduced after menopause in women, but the exact mechanism remains unknown. *Lignsticum chuanxiong* exhibited skin regeneration effects in patients with eczema and psoriasis [43]. SWT inhibited mast cell activation and thus suppressed the inflammatory response, proving useful for alleviation of cutaneous pruritus [6].

**Fig. 3:**
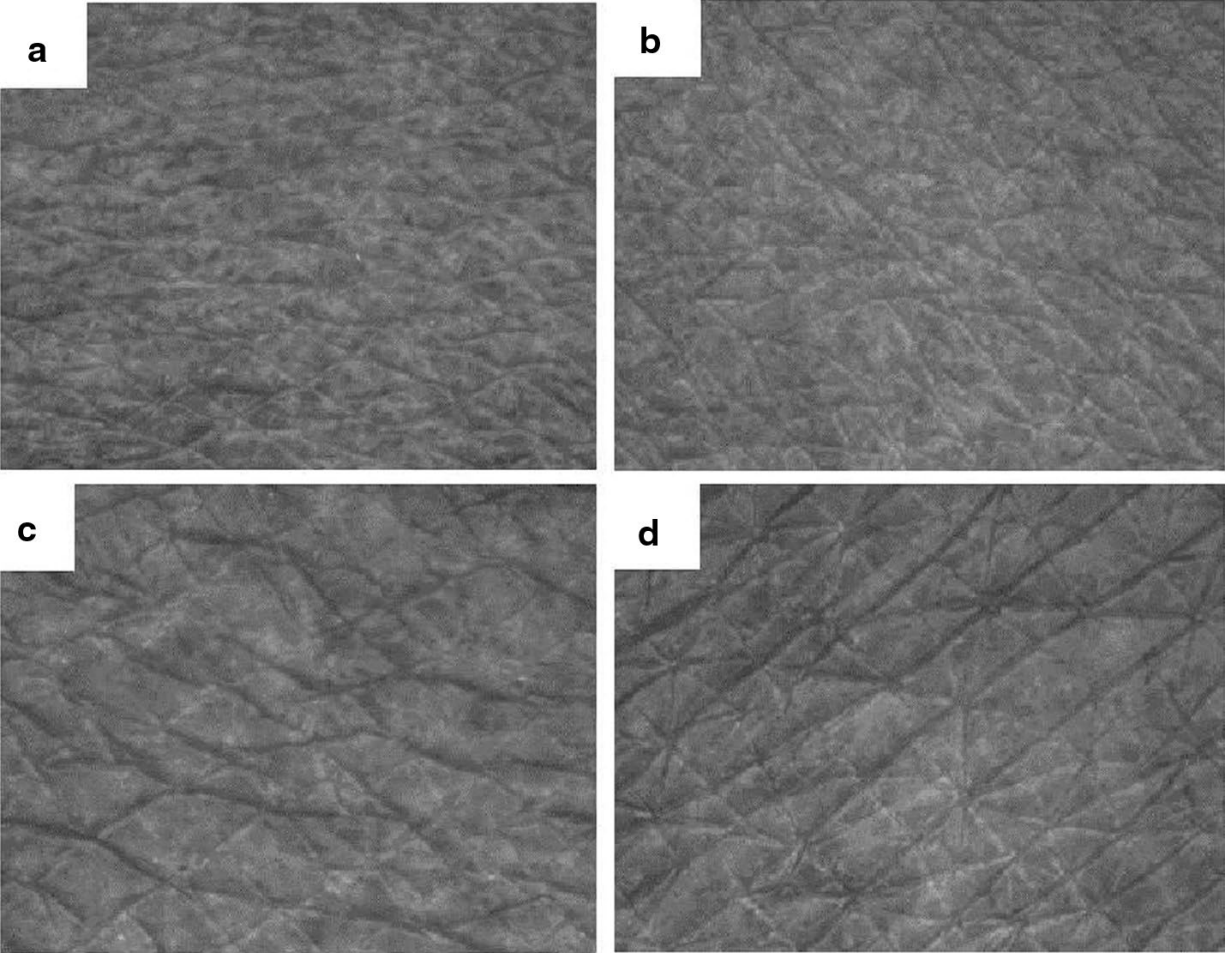
Skin surface topography viewed under ultraviolet light (Visioscan VC 98). Examination of facial and arm skin of participant 23. **a**, **b** represents the facial and arm skin of placebo group, which indicate less skin integrity, whereas **c**, **d** represents the facial and arm skin of 6 month treated SWT group, which indicate *improved skin integrity* and *texture*

Figure 3 depicts the skin surface topography of the facial and arm skin of Participant 23 as viewed under ultraviolet light. Figure 3a, b represents the facial and arm skin of patients in the placebo group, who displayed less skin integrity; Fig. 3c, d shows the facial and arm skin of patients in the SWT group at 6 months, showing improved skin integrity and texture. Elevated levels of free radicals are responsible for the weakening of elastin and collagen, skin wrinkles, fragility, dull appearance, and aging [44]. Because SWT is a good antioxidant, it can effectively scavenge free radicals and thus probably improve skin elasticity and texture. Moreover, cnidilide and ligustilide (*Rhizoma* L.) are present in SWT and may be primarily involved in the inhibition of the skin inflammatory reaction and stimulation of blood circulation by altering sebum secretion [5, 7].

## Conclusions

Oral administration of SWT for 6 months significantly improved the antioxidant level and thereby positively regulated the lipid profile, liver function, and skin integrity and texture.

## Additional files

10.1186/s13020-016-0102-0 Copy of Institutional Review Board approval (provided by Chung Shan Medical University Hospital,Taichung, Taiwan).
10.1186/s13020-016-0102-0 A written informed consent (which were received from all participants before enrollment).
