# Supplementary material for: Antioxidant and physiological effects of Si-Wu-Tang on skin and liver: a randomized, double-blind, placebo-controlled clinical trial
Source: Chin Med. 2016 Jun 30;11:30. doi: 10.1186/s13020-016-0102-0 (PMC4929740; doi:10.1186/s13020-016-0102-0)
Supplement: Supplementary file 1 — 10.1186/s13020-016-0102-0 Copy of Institutional Review Board approval (provided by Chung Shan Medical University Hospital,Taichung, Taiwan). [file 13020_2016_102_MOESM1_ESM.pdf]

中山醫學大學附設醫院人體試驗委員會

Institutional Review Board
Chung Shan Medical University Hospital
No.110, Sec.1, Chien-Kuo N. Road, Taichung, Taiwan 402
Phone: 04-24739595 Fax: 04-23248199

臨床試驗計畫許可書

CSMUH No:CS08008

計畫名稱：漢方滋補飲延緩衰老功能之研究
計劃編號：
計畫主持人：王進崑（中山醫學大學副校長）
版　　本：【Protocol: Version: 2.0, Date:3-Mar-2008 ; Informed Consent Form: Version: 3.0, Date:01-Apr-2008；AD：Version: 2.0, Date:03-Mar-2008】
同意函有效期： 98 年 4 月 13 日

上述計畫已於 97 年 3 月 20 日經本院人體試驗委員會九十七年度第三次會議決議修正後同意，於 97 年 4 月 14 日准予執行。

有關計劃主持人的職責、義務、及注意事項均詳列於背面，請參閱並遵守。

主任委員　韓志平

人體試驗委員會 附設醫院

中　華　民　國　九　十　七　年　四　月　十　四　日

Permission of Clinical Trial
Chung Shan Medical University Hospital

Date: Apr 14, 2008

Protocol Title: Study on the antiaging effect of Chinese herb drink
Protocol No:
Principle Investigator: Chin-Kun Wang
Version: 【Protocol: Version: 2.0, Date:3-Mar-2008 ; Informed Consent Form: Version: 3.0, Date:01-Apr-2008；AD：Version: 2.0, Date:03-Mar-2008】

Above study has been approved by Institutional Review Board of the Chung Shan Medical University Hospital on 14-Apr-2008 and valid through 13-Apr-2009.
About the essential duties, obligations and responsibilities of the principal investigator please refer to the back page.

Chih-Ping Han
Chih-Ping Han, MD/PhD
Chairman
Institutional Review Board

Chung Shan Medical University Hospital Institutional Review Board

附錄二

個人基本資料

姓 名:___________ 性 別: ☐ 男生 ☐女生

年 齡:

身 高: 體脂肪：

體 重: BMI:

目前用藥記錄：

抽煙： ☐ 沒有 ☐ 有

口服避孕藥： ☐ 沒有 ☐ 有

平日是否有服用藥物的習慣: ☐沒有 ☐有

您有吃營養補充劑的習慣: ☐沒有 ☐有

最近一週內是否有感冒或生病而用藥: ☐沒有 ☐有

家族史：☐高血脂症 ☐糖尿病 ☐其他_________

腹部超音波檢查結果:

醫師診察：☐正常 ☐其他

醫師簽名：_____________
