# Supplementary material for: Antioxidant and physiological effects of Si-Wu-Tang on skin and liver: a randomized, double-blind, placebo-controlled clinical trial
Source: Chin Med. 2016 Jun 30;11:30. doi: 10.1186/s13020-016-0102-0 (PMC4929740; doi:10.1186/s13020-016-0102-0)
Supplement: Supplementary file 2 — 10.1186/s13020-016-0102-0 A written informed consent (which were received from all participants before enrollment). [file 13020_2016_102_MOESM2_ESM.pdf]

食品臨床試驗受試者同意書

97.12.21 第七次人體試驗委員會

**計畫名稱：漢方滋補(四物)飲延緩衰老功能之研究**
**執行單位：健康管理學院　　　　　　　　　電話：04-24730022 ext. 11090**<br>**主要主持人：王進崑　　　　　　　　　　　職稱：教授**<br>**協同主持人：楊仁宏　　　　　　　　　　　職稱：醫師**<br>**※二十四小時緊急聯絡人※：賴佳璐　　　　電話：0919683398**
**受試者姓名：**<br>**性別：　　　　　　年齡：**<br>**病歷號碼：**<br>**通訊地址：**<br>**聯絡電話：**<br>**法定代理人姓名（受試者為無行為能力或限制行為能力人)：**<br>**與受試者關係：**<br>**性別：　　　　　　年齡：**<br>**身份證字號：**<br>**通訊地址：**<br>**聯絡電話：**

一、背景介紹與試驗動機：

老化是每個人會面對的問題，而且也是隨時進行的自然生理反應，老化不僅是年齡的增長，更造成生理機能的退化。因此近年來如何有效延緩老化以維持良好生理功能已深受矚目。本計劃主持人過去曾針對老化之臨床研究進行探討，具有良好之經驗。本研究進行所需之漢方滋補飲則由佳格食品股份有限公司提供。

二、試驗的目的及方法：

確認確認漢方滋補飲延緩衰老之功效。主要透過血液生化值與氧化狀態評估與皮膚即肝功能特性確認之。

三、納入及排除條件：

納入：

本試驗將招收 60 位 20~80 歲健康之**男女**性志願參與者，參與之個體需是無服用藥物且無代謝疾病之個體，且經醫師檢查者，並能配合本試驗之進行。受試者分為兩組以交叉試驗進行之，一組給予試驗樣品，另一組則給予安慰劑。

受試婦女資格排除：a. 肝、腎或心臟病者。b. 酗酒者 c. 未控制之糖尿病患 d. 最近一年中風者 e. 精神病或憂鬱症 f. 懷孕、哺乳者。

四、試驗可能產生之副作用及處理對策：

本產品若有產生拉肚子或不適等副作用，受試者將可接受免費醫療協助(由本研究計畫之委託廠商提供)，並隨時終止試驗。

五、預期試驗效果：

漢方滋補(四物)飲具有延緩衰老之功效。

六、試驗進行中受試者之禁忌、限制與應配合之事項：

本試驗將招收 60 位健康之男女性志願參與者，參與之個體需是無服用藥物且無代謝疾病之個體，且經醫師檢查者，並能配合本試驗之進行。個體除進行臨床評估與體位測定外，亦需於試驗前、期間及試驗後進行五次抽血檢查，抽血時間為禁食空腹之上午，每次抽血量為 10ml。

七、機密性：

除主持人外，受試者資料一律密封不外流

八、賠償與保險：

※ 正常服用食品雖安全，如依本研究所訂臨床試驗計劃，因而發生傷害，由佳格食品股份有限公司（試驗委託者機構或廠商/若無委託機構，請填寫計畫主持人）負擔責任，依法賠償。由佳格食品股份有限公司（試驗委託者廠商機構/若無委託機構，請填寫計畫主持人本人）提供本試驗相關訊息暨諮詢。（註：試驗委託者應負責試驗主持人或試驗機構因試驗所生之賠償責任或投保責任保險。）

※ 除法定賠償及醫療照顧外，本研究不提供其他形式之賠償或補償。**若您不願意接受這樣的風險，您有權選擇不參與這項試驗。**

九、參加試驗個人權益之保護：

A. 受試者參加本試驗皆不須繳交額外費用。

B. 試驗過程中，與你(妳)的健康或是疾病有關，可能影響你(妳)繼續接受臨床試驗意願的任何重大發現，都將即時提供給你(妳)。

C. 如果你(妳)在試驗過程中對試驗工作性質產生疑問，對身為患者之權利有意見或懷疑因參與研究而受害時，可與本院人體試驗委員會施小姐聯絡請求諮詢，其電話號碼為：04-24739595 轉 34115。

D. 為進行試驗工作，你(妳)必須接受 **楊仁宏** 醫師的照顧。如果你(妳)現在或於試驗期間有任何問題或狀況，請不必客氣，可與在 中山醫院 **皮膚科** 部的 楊仁宏 （全天二十四小時皆可聯繫電話；**0921329093**）醫師聯絡。（**若試驗主持人非直接從事醫療業務之醫師，亦可更改為可全天二十四小時皆可聯繫之試驗主持人或試驗團隊成員，但需有義務協助受試者並提供必要之就醫援助**）

E. 主持人已將同意書副本交給你(妳)，並已完整說明本研究之性質與目的。王進崑 主持人已回答您有關食品與研究的問題。

十、試驗之退出與中止：

受試者可自由決定是否參加本試驗，並於試驗過程中可隨時撤銷同意，退出試驗，不須任何理由，且不會引起任何不愉快或影響其日後醫師對受試者的醫療照顧。此外，受試者並已充份了解試驗主持人或贊助廠商亦可能於必要時中止該試驗之進行。

十一、其它：

無。

十二、受試者同意書需經由受試者或其法定代理人的簽署，並載明日期始生效力：

A. 主要主持人、協同主持人或代理主持人已詳細解釋有關本研究計畫中上述研究方法的性質與目的，及可能產生的危險與利益。

主要主持人/協同主持人/代理主持人簽章：

日期：□□□□年□□月□□日

B. 受試者已詳細瞭解上述研究方法及其所可能產生的危險與利益，有關本試驗計畫的疑問，業經計畫主持人詳細予以解釋。本人同意接受為臨床試驗計畫的自願受試者。（受試者為無行為能力人者，由法定代理人代為之；受試者為限制行為能力人者，應得法定代理人之同意；受試者雖非無行為能力或限制行為能力者，但因無意識或精神錯亂無法自行為之時，由有同意權之人為之。前項有同意權人為配偶及同居之親屬。）

受試者簽章：

（法定）代理人或有同意權人簽章：

關係：

身份證字號：□□□□□□□□□□ 聯絡電話：□□□□□□□□□□

通訊地址：

簽章： 日期：□□□□年□□月□□日

C. 受試者、法定代理人或有同意權之人皆無法閱讀時，應由見證人在場參與所有有關受試者同意書之討論。見證人應閱讀受試者同意書及提供受試者之任何其他書面資料，以見證試驗主持人或其指定之人員已經確切地將其內容向受試者、法定代理人或有同意權之人為解釋，並確定其充分了解所有資料之內容。第一項情形，受試者、法定代理人或有同意權之人，仍應於受試者同意書親筆簽名並載明日期。但得以指印代替簽名。見證人於完成第二項之行為，並確定受試者、法定代理人或有同意權之人之同意完全出於其自由意願後，應於受試者同意書簽名並載明日期。試驗相關人員不得為見證人。

1. 見證人簽章：

姓名：

身份證字號：□□□□□□□□□□ 聯絡電話：□□□□□□□□□□

通訊地址：

簽章： 日期：□□□□年□□月□□日
